# Complications of Percutaneous Release of the Trigger Finger

**DOI:** 10.7759/cureus.4132

**Published:** 2019-02-25

**Authors:** Alper Aksoy, Emin Sir

**Affiliations:** 1 Plastic Surgery, Konur Hospital, Bursa, TUR; 2 Plastic Surgery, Private Hospital, Izmir, TUR

**Keywords:** trigger finger, percutaneous release operations, complications, needle, pulley, hand surgery, tendon laceration, open surgery, stenosing tenosynovitis, digital nerve damage

## Abstract

Aim: Trigger finger is a common cause of hand pain and dysfunction. In this study, we aimed to evaluate retrospectively short and long-term outcomes of patients with trigger fingers who underwent percutaneous release operations.

Materials and methods: Thirty-nine patients who underwent percutaneous release of the trigger finger were analyzed retrospectively. The patients were evaluated for digital nerve injury (hypoesthesia), recurrence, painful scar, and tendon rupture.

Results: The patients' median age was 54 years (minimum 32 years - maximum 63 years). Hypoesthesia was most frequently seen at the first and fourth fingers. At the end of the first year, one patient developed tendon rupture (fourth finger). Recurrences were seen at the end of the first (n=5) and third (n=9) years. Recurrence was mostly seen in the fourth finger, followed by the third finger. Painful scars were observed in two patients.

Conclusion: Percutaneous release is a blindly performed intervention and the emergence of unexpected complications should not be forgotten.

## Introduction

Trigger finger is a common cause of hand pain and dysfunction in the adult population [1]. It is a stenosing flexor tenosynovitis of the fingers and thumb as a result of repetitive use [1,2]. Treatment of trigger finger usually is conservative if it is uncomplicated and if it has a short history of symptoms. It includes steroid injections and splinting [2,3,4]. Percutaneous trigger finger release is simple and effective with success rates of 84% to 100% at the mid-term follow-up [4,5,6]. In trigger finger surgery, exposure of the A1 pulley after mini-incision and then its longitudinal excision to release the tendon is the most prevalently known method. However, this procedure is a classical procedure, which requires an incision (though a small one) and its closure [5,7]. The percutaneous release procedure developed by Eastwood et al. has been popularized, suggesting its ease of application, low-cost, and decreased complication rates [8]. Numerous publications related to these two surgical interventions are found in the literature. Some authors have indicated that despite apparently improved clinical outcomes of the percutaneous release procedure, the A1 pulley cannot be completely excised, with a possibility of causing longitudinal wound scar, increased probability of nerve injury, and rates of recurrence, which constitute the disadvantages of this approach [7,9].

In the treatment of trigger finger, the first alternative is application of conservative methods [8]. In cases of failed conservative treatment, surgery can solve this problem in the majority of the cases. Surgery constitutes open surgery and in recent years via the more popularized percutaneous method. Both methods have advantages and disadvantages over one another. In this study, we aimed to evaluate retrospectively short- and long-term outcomes of patients with trigger fingers on whom we applied percutaneous release operations.

## Materials and methods

We applied percutaneous release of 39 fingers of 39 patients with a diagnosis of trigger finger (27 female, 12 male; median age, 54 years) between January 2014 and December 2018. The distribution of patients based on laterality of trigger fingers is given in Table 1. Before the intervention, the median duration of the patients’ complaints was seven months (6-12 months). All patients underwent conservative treatment preoperatively. All patients were in Green Grade 3. Percutaneous release was performed under operating room conditions using local anesthesia applied with a 21 G syringe needle. After excision, active and passive finger movements were controlled and the ease of performing finger movements was observed. After the intervention, splint was not applied and elevation was described and oral non steroidal anti-inflammatory drugs (NSAIDs) were prescribed to be used in case of need. The patients were sent home the same day. Intermittent ice pack application on the incision site for six hours was described to the patients in order to decrease inflammation and potential development of edema. The patients returned to their daily activities after an average of one to two days. The patients were invited to attend control visits at the first month, first and third years, postoperatively. The patients were evaluated for digital nerve injury (hypoesthesia), recurrence, painful scar, and tendon rupture.

**Table 1:** Side distribution of the fingers.

Finger	Right	Left
Thumb	3	5
Index finger	4	3
Middle finger	6	3
Ring finger	7	5
Little finger	1	2

## Results

At the end of the evaluation process of 39 patients with Grade 3 trigger finger who underwent percutaneous release procedures, hypoesthesia was found at the end of the postoperative first (n=7) and third years (n=2). Hypoesthesia was most frequently seen at the first and fourth fingers. At the end of the third year, hypoesthesia was observed involving the same fingers. At the end of the first year, one patient developed tendon rupture (fourth finger) and at the end of the third year no evidence of rupture was observed. Recurrences were seen at the end of the first (n=5) and third (n=9) years. Recurrence was mostly seen in the fourth finger, followed by the third finger. Painful scars were observed in two patients, while at the end of the third year no painful scar was seen. The distribution of painful scars among fingers is shown in Table 2. Wound superficial infection was not observed in any patient.

**Table 2:** First and third year complications according to fingers.

	First year					Third year				
	Thumb	Index finger	Middle finger	Ring finger	Little finger	Thumb	Index finger	Middle finger	Ring finger	Little finger
Hypoesthesia/ digital nerve injury	3	1	1	2	0	1	0	0	1	0
Tendon rupture	0	0	1	0	0	0	0	0	0	0
Recurrence	0	1	1	3	0	1	1	3	4	0
Scar	1	0	0	1	0	0	0	0	0	0

## Discussion

Trigger finger is characterized by the thickening of the flexor tendons of fingers or nodule formation resulting in the derangement of the communication between the tendon and its sheath at the level of the head of the metacarpal. In other words, it is due to thickening of the tendon sheath and the A1 pulley and sometimes nodule formation on the tendon together with luminal narrowing [10]. On physical examination, consolidation of the synovial tissue under the A1 pulley and a solid nodule shifting its place with the tendon is palpated. The nodule is generally localized at the level of the metacarpophalangeal joint where the tendon enters into proximal annulus. However, in patients with rheumatoid arthritis, these locations may be numerous and vary. This nodule creates a snapping sensation when it passes underneath the pulley. In newly developed cases this movement causes pain. In later stages the nodule can still be palpated, but this snapping sensation and pain can become milder. During movements of fingers, pain can be elicited. In the community, trigger finger can be seen in association with recurrent trauma, diabetes mellitus, rheumatoid arthritis, carpal tunnel syndrome, Dupuytren's contracture, amyloidosis, hypothyroidism, mucopolysaccharidoses, and congestive heart disease. Diagnosis is made based on medical history and physical examination. It usually affects the first, third, and fourth fingers and it is usually painless. In diabetic patients, more frequently, more than one finger is affected.

In the conservative treatment of trigger finger, injections of steroids and local anesthetics and splint application are first prescribed. In patients who did not benefit from conservative treatment, surgery is performed. The choice of surgical intervention has not been definitively decided upon, and debates between the alternatives of open surgery and the percutaneous release procedure are still continuing. The percutaneous release procedure developed by Eastwood et al. has been popularized with assertions favoring its ease of application and lower cost and complication rates. [8]. Also, conservative treatment was applied to all patients before surgical procedure in this study. In surgical release procedures, problems encountered can be easily solved safely in 97%-100% of the cases. Physicians advocating the percutaneous release procedure indicated relevant complications as incision-related infection, formation of painful scars, bowstringing of flexor tendons due to pulley injury, joint nodules, weakness, and digital nerve-artery damage, and they intended to avoid these complications.

However, in cadaver studies, small longitudinal ruptures were detected on flexor tendons after percutaneous release procedures. After percutaneous release procedures, abrasive type injuries have been reported. Pope et al. indicated that during percutaneous release procedures, 10%-15% of the distal part of the pulley could not be excised [11]. Cebesoy et al. performed percutaneous release surgery on 25 fingers, four of which required open surgery because of restricted digital movements one week later [12]. Still we observed recurrences in five and nine patients at the end of the first and three years, respectively. In patients who underwent open surgery, the pulleys were covered with fibrous scar tissue. As indicated by Pope et al., we think that this scar tissue may be due to the remaining distal segment or incomplete excision [11]. Since this procedure was mostly performed for the fourth finger, recurrence rates were more frequently observed in this finger. The most dreadful major complication of the percutaneous technique is tendon rupture. In studies on cadavers, tendon lacerations were observed after percutaneous release procedures [13]. These lacerations cause weakening of tendons and they can lead to the development of tendon ruptures in the short- and long-term. Only in one of our patients who underwent percutaneous trigger finger release operation, tendon rupture was observed at the end of the first postoperative year. However, at the end of the third year tendon rupture was not detected.

Painful tenosynovitis can be seen due to the laceration of the flexor tendon after application of the percutaneous technique [14]. These cases can benefit from corticosteroid injections. We did not observe any episode of painful tenosynovitis.

One of the most important complications of the percutaneous treatment of trigger finger is digital nerve injury [13]. In open surgery, release procedure is performed after exposure of the nerve, which is protected from trauma; however, percutaneous surgical procedure is performed blindly. In a cadaver study performed by Buldu et al., the authors investigated the potential complications that might occur during open and percutaneous surgery applied for trigger finger and indicated that in open surgery, the digital nerve distal to the metacarpophalangeal joint is at a risk of injury and therefore interphalangeal and palmar creases should be given due importance [15]. To avoid nerve injury, generally percutaneous release is not recommended for thumb and index finger because of their close vicinity to nervous tissue [13]. In our study, hypoesthesia was observed at the end of the first (n=7) and third (n=2) postoperative years. All of these patients declined operation. They indicated that hypoesthesia did not affect their standard of life.

Bain et al. recommended percutaneous release operation for active and movable trigger fingers and discouraged application of this procedure for locked fingers or those with tenosynovitis [13]. In cases that presented with chronic locked fingers, flexion contracture can be observed. In this case open surgery is a must, and immediately following open surgery hand rehabilitation is required during the early phase.

In a study by Lange-Rieß et al. on success rates in open surgery performed on 350 cases in two series, the authors detected only nine perioperative complications as superficial wound infection (n=2), one delayed wound healing (n=1), and transient sensory lesion of the digital nerve (n=6), and at the end of a median follow-up of 14 years no permanent complication was observed [16]. Ertem et al. detected transient scar sensitivity in a series of 17 cases. Among their cases, superficial wound infection and delayed wound healing due to postoperative hematoma was observed in one patient [17]. We did not detect any wound infection in our cases.

## Conclusions

In this retrospective study we found that hypoesthesia was the most frequently seen complication, and in the long-term, recurrence was also a frequent complication after the trigger finger percutaneous release procedure. The percutaneous method and open surgery are the existing surgical interventions and various procedural and post-procedural complications can be observed. However, percutaneous release is a blindly performed intervention and the emergence of unexpected complications should not be forgotten.

## References

[1] Matthews A, Smith K, Read L, Nicholas J, Schmidt E (2019). Trigger finger: an overview of the treatment options. JAAPA.

[2] Chao M, Wu S, Yan T (2009). The effect of miniscalpel-needle versus steroid injection for trigger thumb release. J Hand Surg Eur.

[3] Jegal M, Woo SJ, Lee H Il, Shim JW, Park MJ (2018). Effects of simultaneous steroid injection after percutaneous trigger finger release: a randomized controlled trial. J Hand Surg Eur Vol.

[4] Ng WKY, Olmscheid N, Worhacz K, Sietsema D, Edwards S (2018). Steroid injection and open trigger finger release outcomes: a retrospective review of 999 digits. Hand (N Y).

[5] Zhao JG, Kan SL, Zhao L, Wang ZL, Long L, Wang J, Liang CC (2014). Percutaneous first annular pulley release for trigger digits: a systematic review and meta-analysis of current evidence. J Hand Surg Am.

[6] Park MJ, Oh I, Ha KI (2004). A1 pulley release of locked trigger digit by percutaneous technique. J Hand Surg Br.

[7] Ertürk E, Altay Ma, Kalander AM (Derg). Mini açık tetik parmak gevşetmesi. [Article in Turkish]. Tıp Arast.

[8] Eastwood DM, Gupta KJ, Johnson DP (1992). Percutaneous release of the trigger finger: an office procedure. J Hand Surg.

[9] (2005). Tenosinovitis. Green’s Operative Hand Surgery.

[10] Kılıç BA, Kıter AE, Selçuk Y (2002). Tetik parmak tedavisinde perkütan cerrahi girişimin normal anatomik yapılara etkisi. [Article in Turkish]. Acta Orthop Trauma.

[11] Pope DF, Wolfe SW (1995). Safety and efficacy of percutaneous trigger finger release. J Hand Surg.

[12] Cebesoy O (2006). Percutaneous trigger finger treatment. Tech Hand Up Extrem Surg.

[13] Bain GI, Turnbull J, Charles MN, Roth JH, Richards RS (1995). Percutaneous A1 pulley release: a cadaveric study. J Hand Surg Am.

[14] Wojahn RD, Foeger NC, Gelberman RH, Calfee RP (2014). Long-term outcomes following a single corticosteroid injection for trigger finger. J Bone Joint Surg Am.

[15] Buldu H, Cepel S, Ki N, Ağritmiş H (2006). References to avoid complications in releases of the trigger thumb: a cadaveric study. [Article in Turkish]. Acta Orthop Traumatol Turc.

[16] Lange-Riess D, Schuh R, Hönle W, Schuh A (2009). Long-term results of surgical release of trigger finger and trigger thumb in adults. Arch Orthop Trauma Surg.

[17] Ertem K, Inan M, Coskun H, Bora A (2003). Minimal açık insizyonla gevşetme yapılan tetik parmaklı hastalardaki cerrahi tedavi sonuçlarımız. [Article in Turkish]. Inönü Univ Tıp Fak Derg.

